# 1,3-Bis(3-phenyl­prop­yl)-1*H*-1,3-benzimidazole-2(3*H*)-selone

**DOI:** 10.1107/S1600536811013985

**Published:** 2011-04-22

**Authors:** Mehmet Akkurt, Ülkü Yılmaz, Hasan Küçükbay, Orhan Büyükgüngör

**Affiliations:** a Department of Physics, Faculty of Sciences, Erciyes University, 38039 Kayseri, Turkey; b Department of Chemistry, Faculty of Arts and Sciences, Ínönü University, 44280 Malatya, Turkey; c Department of Physics, Faculty of Arts and Sciences, Ondokuz Mayıs University, 55139 Samsun, Turkey

## Abstract

The title mol­ecule, C_25_H_26_N_2_Se, has mirror symmetry, with the mirror plane passing through the atoms of the C=Se bond and the mid-points of the two C—C bonds of the benzene ring of the benzimidazole group. The dihedral angle between the benzimidazole ring system and the phenyl ring is 71.62 (14)°.

## Related literature

For general background to benzimidazole derivatives, see: Aydın *et al.* (1998 ▶); Böhm & Herrmann (2000 ▶); Küçükbay *et al.* (1996 ▶, 1997 ▶); Lappert *et al.* (2009 ▶); Wanzlick & Schikora (1960 ▶); Yıldırım *et al.* (2006 ▶); Yılmaz & Küçükbay (2009 ▶); Çetinkaya *et al.* (1994 ▶, 1998 ▶). For related structures, see: Akkurt *et al.* (2004 ▶); Aydın *et al.* (1999 ▶); Yalçın *et al.* (2008 ▶).
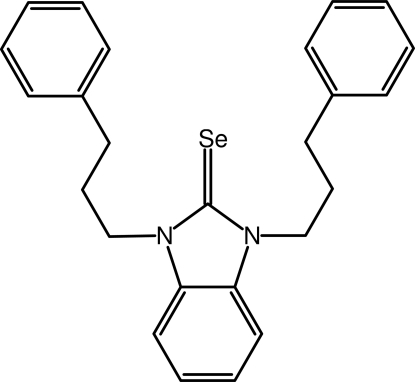


## Experimental

### 

#### Crystal data

C_25_H_26_N_2_Se
*M* *_r_* = 433.44
Tetragonal, 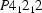

*a* = 10.5150 (3) Å
*c* = 19.8142 (8) Å
*V* = 2190.76 (13) Å^3^
*Z* = 4
Mo *K*α radiation
μ = 1.73 mm^−1^
*T* = 296 K
0.68 × 0.58 × 0.52 mm

#### Data collection

Stowe IPDS 2 diffractometer
Absorption correction: integration (*X-RED32*; Stoe & Cie, 2002 ▶) *T*_min_ = 0.322, *T*_max_ = 0.408
16780 measured reflections
2531 independent reflections
2225 reflections with *I* > 2σ(*I*)
*R*_int_ = 0.055

#### Refinement

*R*[*F* ^2^ > 2σ(*F* ^2^)] = 0.032
*wR*(*F* ^2^) = 0.066
*S* = 1.07
2531 reflections
128 parameters
H-atom parameters constrained
Δρ_max_ = 0.17 e Å^−3^
Δρ_min_ = −0.24 e Å^−3^
Absolute structure: Flack (1983 ▶), 1003 Freidel pairs
Flack parameter: 0.004 (12)

### 

Data collection: *X-AREA* (Stoe & Cie, 2002 ▶); cell refinement: *X-AREA*; data reduction: *X-RED32* (Stoe & Cie, 2002 ▶); program(s) used to solve structure: *SIR97* (Altomare *et al.*, 1999 ▶); program(s) used to refine structure: *SHELXL97* (Sheldrick, 2008 ▶); molecular graphics: *ORTEP-3* (Farrugia, 1997 ▶); software used to prepare material for publication: *WinGX* (Farrugia, 1999 ▶).

## Supplementary Material

Crystal structure: contains datablocks global, I. DOI: 10.1107/S1600536811013985/qm2004sup1.cif

Structure factors: contains datablocks I. DOI: 10.1107/S1600536811013985/qm2004Isup2.hkl

Additional supplementary materials:  crystallographic information; 3D view; checkCIF report

## Figures and Tables

**Fig. 1.**
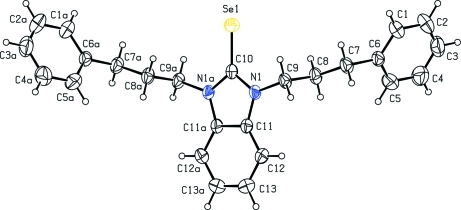
View of the title molecule, showing the atom labelling scheme. Displacement ellipsoids for non-H atoms are drawn at the 30% probability level. (Symmetry code: (a) y, x, 1 - z).

**Fig. 2.**
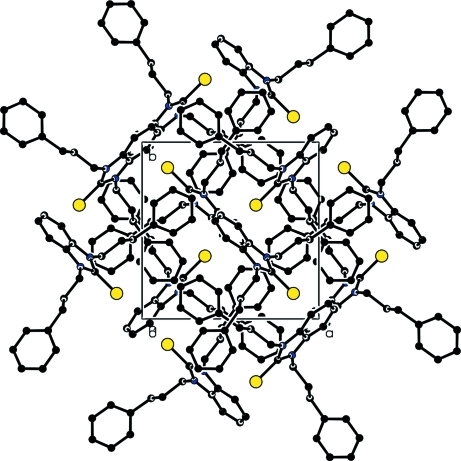
The packing diagram of (I) viewing down the b axis. All hydrogen atoms have been omitted for clarity.

## supplementary crystallographic information

### Comment

Electron-rich olefins (EROs) have been attracted considerable attention in both organic and inorganic preparative literature due to their unique properties as reagent and reaction intermediates since their first report by Wanzlick in 1960 (Wanzlick & Schikora, 1960; Böhm & Herrmann, 2000).

Benzimidazolium salts are convenient precursors for EROs through reacting with a strong base such as NaH comparing with other methods such as reacting a secondary amine (*N*,*N*'-disubstituted-1,2-diaminobenzene) with an acetal, chloral or triethyl orthoformate. We have synthesized and isolated first time the ERO, bis(1,3-dimethybenzimidazolidine-2-ylidene) (Çetinkaya *et al.*, 1994). We have also synthesized a number of EROs using different synthesis methods and used them to synthesize many organic or organometallic compounds (Küçükbay *et al.*, 1996, Küçükbay *et al.*, 1997, Çetinkaya *et al.*, 1998; Aydın *et al.*, 1998 Yıldırım *et al.*, 2006; Yılmaz & Küçükbay, 2009). Their electron-richness confers on them a very high reactivity as strong nucleophiles, which assist in the preparation of numerous products by reaction, amongst others, with group 16 elements, transition metals, and many protic compounds (Lappert *et al.*, 2009). It is known that the ultimate oxidation product of EROs with air is urea; sulfur, selenium and tellurium react similarly to give the corresponding analogues. The objective of the present study was to elucidate the crystal structure of the title compound which is new ERO derivative.

In the title molecule (I), Fig. 1, the Se═C bond length is 1.828 (2) Å, and this value is similar to those [1.829 (3) Å] found in 1-ethyl-3-(2-phenylethyl)benzimidazole-2-selone (Akkurt *et al.*, 2004) and [1.825 (7) Å] found in 1,3-dimethylbenzimidazole-2-selone (Aydın *et al.*, 1999), and is shorter than that [2.058 (4) Å] found for the Te═ C bond length in 1,3-bis(3-phenylpropyl)1*H*-benzimidazole- 2(3*H*)-tellurone (Yalçın *et al.*, 2008).

The molecular structure is stabilized by a weak C—H···Se interaction (Table 1). The benzimidazole ring system (N1/C10/C11/C12/C13/N1a/C11*a*/C12*a*/C13*a*) of (I) is planar (r.m.s deviation of fitted atoms is 0.09 (3) Å). The dihedral angle between the phenyl ring (C1–C6) and the benzimidazole ring is 71.62 (14)°. The molecular packing in (I) is shown in Fig. 2.

### Experimental

A mixture of bis(1,3-di(3-phenylpropyl)benzimidazolidine-2-ylidene) (0.68 g, 0.96 mmol) and selenium (0.15 g, 1.90 mmol) in dry toluene (10 ml) was heated under reflux for 2 h. Then the mixture was filtered to remove unreacted selenium and all volatiles were removed *in vacuo* (0.02 m mH g). The crude product was crystallized from alcohol upon cooling to 243 K. Yield: 0.63 g, 76%; m.p.: 439–441 K; *v*_(CSe)_= 1480 cm^-1^. Anal. found: C 69.58, H 5.98, N 6.38%. Calculated for C_25_H_26_N_2_Se: C 69.27, H 6.05, N 6.46%. ^1^H-NMR (δ, CDCl_3_): 7.26–7.06 (m, 14H, Ar—H), 4.47 (t, 4H, N*CH_2_*CH_2_CH_2_C_6_H_5_, *J* = 7.8 Hz), 2.81 (t, 4H, NCH_2_CH_2_*CH_2_*C_6_H_5_, *J* = 7.8 Hz), 2.23 (quint, 4H, NCH_2_*CH_2_*CH_2_C_6_H_5_, *J* = 7.8 Hz). ^13^C-NMR (δ, CDCl_3_): 165.7 (C=Se), 140.8, 132.9, 128.5, 128.4, 126.2, 123.2 and 109.5 (Ar-*C*), 46.1 (N*CH_2_*CH_2_CH_2_C_6_H_5_), 33.0 (NCH_2_CH_2_*CH_2_*C_6_H_5_), 29.3 (NCH_2_*CH_2_*CH_2_C_6_H_5_).

### Refinement

All H atoms were positioned geometrically with C—H = 0.93–0.97 Å, and refined using a riding model with *U*_iso_(H) = 1.2*U*_eq_(C). The absolute configuration of the title compound was established by refinement of the Flack (1983) parameter.

### Crystal data

**Table:**

C_25_H_26_N_2_Se	*D*_x_ = 1.314 Mg m^−^^3^
*M**_r_* = 433.44	Mo *K*α radiation, λ = 0.71073 Å
Tetragonal, *P*4_1_2_1_2	Cell parameters from 20954 reflections
Hall symbol: P 4abw 2nw	θ = 1.9–28.0°
*a* = 10.5150 (3) Å	µ = 1.73 mm^−^^1^
*c* = 19.8142 (8) Å	*T* = 296 K
*V* = 2190.76 (13) Å^3^	Block, colourless
*Z* = 4	0.68 × 0.58 × 0.52 mm
*F*(000) = 896	

### Data collection

**Table:**

Stowe IPDS 2 diffractometer	2531 independent reflections
Radiation source: sealed X-ray tube, 12 x 0.4 mm long-fine focus	2225 reflections with *I* > 2σ(*I*)
plane graphite	*R*_int_ = 0.055
Detector resolution: 6.67 pixels mm^-1^	θ_max_ = 27.5°, θ_min_ = 2.2°
ω scans	*h* = −13→13
Absorption correction: integration (*X-RED32*; Stoe & Cie, 2002)	*k* = −13→13
*T*_min_ = 0.322, *T*_max_ = 0.408	*l* = −25→25
16780 measured reflections	

### Refinement

**Table:**

Refinement on *F*^2^	Secondary atom site location: difference Fourier map
Least-squares matrix: full	Hydrogen site location: inferred from neighbouring sites
*R*[*F*^2^ > 2σ(*F*^2^)] = 0.032	H-atom parameters constrained
*wR*(*F*^2^) = 0.066	*w* = 1/[σ^2^(*F*_o_^2^) + (0.0285*P*)^2^ + 0.3345*P*] where *P* = (*F*_o_^2^ + 2*F*_c_^2^)/3
*S* = 1.07	(Δ/σ)_max_ < 0.001
2531 reflections	Δρ_max_ = 0.17 e Å^−^^3^
128 parameters	Δρ_min_ = −0.24 e Å^−^^3^
0 restraints	Absolute structure: Flack (1983), 1003 Freidel pairs
Primary atom site location: structure-invariant direct methods	Flack parameter: 0.004 (12)

### Special details

**Table:**

Geometry. Bond distances, angles *etc*. have been calculated using the rounded fractional coordinates. All su's are estimated from the variances of the (full) variance-covariance matrix. The cell e.s.d.'s are taken into account in the estimation of distances, angles and torsion angles
Refinement. Refinement on *F*^2^ for ALL reflections except those flagged by the user for potential systematic errors. Weighted *R*-factors *wR* and all goodnesses of fit *S* are based on *F*^2^, conventional *R*-factors *R* are based on *F*, with *F* set to zero for negative *F*^2^. The observed criterion of *F*^2^ > σ(*F*^2^) is used only for calculating -*R*-factor-obs *etc*. and is not relevant to the choice of reflections for refinement. *R*-factors based on *F*^2^ are statistically about twice as large as those based on *F*, and *R*-factors based on ALL data will be even larger.

### Fractional atomic coordinates and isotropic or equivalent isotropic displacement parameters (Å^2^)

**Table:**

	*x*	*y*	*z*	*U*_iso_*/*U*_eq_	
Se1	0.35581 (2)	0.35581 (2)	0.50000	0.0563 (1)	
N1	0.20501 (16)	0.15139 (19)	0.55133 (8)	0.0486 (5)	
C1	0.6763 (3)	0.0124 (4)	0.71439 (17)	0.0933 (12)	
C2	0.7825 (3)	−0.0624 (5)	0.7188 (2)	0.116 (2)	
C3	0.7813 (3)	−0.1823 (4)	0.69632 (19)	0.0997 (16)	
C4	0.6739 (4)	−0.2301 (4)	0.6684 (2)	0.1013 (16)	
C5	0.5668 (3)	−0.1541 (3)	0.66230 (17)	0.0868 (11)	
C6	0.5656 (2)	−0.0317 (3)	0.68586 (12)	0.0593 (8)	
C7	0.4485 (2)	0.0505 (3)	0.68283 (11)	0.0623 (9)	
C8	0.3849 (2)	0.0578 (3)	0.61434 (11)	0.0587 (8)	
C9	0.2714 (2)	0.1457 (3)	0.61606 (10)	0.0544 (7)	
C10	0.2329 (2)	0.2329 (2)	0.50000	0.0474 (6)	
C11	0.1061 (2)	0.0716 (2)	0.53275 (11)	0.0508 (7)	
C12	0.0438 (3)	−0.0248 (3)	0.56674 (13)	0.0675 (9)	
C13	−0.0536 (3)	−0.0863 (3)	0.53297 (16)	0.0813 (11)	
H1	0.67910	0.09500	0.73110	0.1120*	
H2	0.85650	−0.02940	0.73770	0.1390*	
H3	0.85380	−0.23260	0.69990	0.1200*	
H4	0.67210	−0.31380	0.65320	0.1220*	
H5	0.49430	−0.18690	0.64180	0.1040*	
H7A	0.47150	0.13590	0.69670	0.0750*	
H7B	0.38720	0.01830	0.71520	0.0750*	
H8A	0.44560	0.08840	0.58120	0.0700*	
H8B	0.35770	−0.02650	0.60080	0.0700*	
H9A	0.21270	0.11680	0.65050	0.0650*	
H9B	0.29950	0.23050	0.62830	0.0650*	
H12	0.06640	−0.04730	0.61050	0.0810*	
H13	−0.09810	−0.15080	0.55470	0.0980*	

### Atomic displacement parameters (Å^2^)

**Table:**

	*U*^11^	*U*^22^	*U*^33^	*U*^12^	*U*^13^	*U*^23^
Se1	0.0586 (1)	0.0586 (1)	0.0515 (2)	−0.0076 (2)	0.0010 (1)	−0.0010 (1)
N1	0.0503 (9)	0.0574 (10)	0.0382 (8)	0.0010 (9)	−0.0077 (7)	0.0101 (8)
C1	0.070 (2)	0.097 (2)	0.113 (2)	−0.0031 (16)	−0.0375 (17)	0.0133 (19)
C2	0.063 (2)	0.135 (4)	0.151 (4)	0.003 (2)	−0.040 (2)	0.024 (3)
C3	0.064 (2)	0.127 (3)	0.108 (3)	0.021 (2)	−0.0043 (18)	0.044 (2)
C4	0.086 (3)	0.088 (2)	0.130 (3)	0.0157 (19)	−0.001 (2)	0.018 (2)
C5	0.0574 (16)	0.087 (2)	0.116 (2)	−0.0015 (16)	−0.0133 (16)	0.005 (2)
C6	0.0501 (13)	0.0737 (17)	0.0540 (13)	−0.0025 (12)	−0.0056 (11)	0.0214 (12)
C7	0.0573 (14)	0.0798 (18)	0.0498 (12)	0.0021 (13)	−0.0118 (11)	0.0089 (12)
C8	0.0570 (15)	0.0762 (16)	0.0429 (11)	0.0077 (12)	−0.0055 (9)	0.0103 (10)
C9	0.0543 (12)	0.0730 (15)	0.0358 (9)	0.0014 (13)	−0.0081 (9)	0.0081 (12)
C10	0.0517 (10)	0.0517 (10)	0.0389 (13)	0.0066 (13)	−0.0040 (10)	0.0040 (10)
C11	0.0520 (14)	0.0536 (13)	0.0467 (11)	0.0019 (11)	−0.0064 (9)	0.0096 (10)
C12	0.0673 (16)	0.0664 (16)	0.0689 (15)	−0.0040 (12)	−0.0058 (13)	0.0236 (13)
C13	0.075 (2)	0.0690 (19)	0.100 (2)	−0.0168 (17)	−0.0071 (16)	0.0207 (16)

### Geometric parameters (Å, °)

**Table:**

Se1—C10	1.828 (2)	C12—C13	1.384 (4)
N1—C9	1.462 (3)	C13—C13^i^	1.394 (4)
N1—C10	1.362 (2)	C1—H1	0.9300
N1—C11	1.386 (3)	C2—H2	0.9300
C1—C2	1.369 (5)	C3—H3	0.9300
C1—C6	1.375 (4)	C4—H4	0.9300
C2—C3	1.337 (7)	C5—H5	0.9300
C3—C4	1.354 (5)	C7—H7A	0.9700
C4—C5	1.386 (5)	C7—H7B	0.9700
C5—C6	1.369 (4)	C8—H8A	0.9700
C6—C7	1.506 (4)	C8—H8B	0.9700
C7—C8	1.515 (3)	C9—H9A	0.9700
C8—C9	1.510 (4)	C9—H9B	0.9700
C11—C12	1.382 (4)	C12—H12	0.9300
C11—C11^i^	1.396 (3)	C13—H13	0.9300
			
C9—N1—C10	125.33 (18)	C2—C3—H3	120.00
C9—N1—C11	124.52 (19)	C4—C3—H3	120.00
C10—N1—C11	110.13 (16)	C3—C4—H4	120.00
C2—C1—C6	121.6 (4)	C5—C4—H4	120.00
C1—C2—C3	120.9 (3)	C4—C5—H5	119.00
C2—C3—C4	119.6 (3)	C6—C5—H5	119.00
C3—C4—C5	119.9 (4)	C6—C7—H7A	108.00
C4—C5—C6	121.3 (3)	C6—C7—H7B	108.00
C1—C6—C5	116.7 (3)	C8—C7—H7A	108.00
C1—C6—C7	121.0 (3)	C8—C7—H7B	108.00
C5—C6—C7	122.3 (2)	H7A—C7—H7B	108.00
C6—C7—C8	115.2 (2)	C7—C8—H8A	109.00
C7—C8—C9	111.1 (2)	C7—C8—H8B	109.00
N1—C9—C8	112.51 (19)	C9—C8—H8A	109.00
Se1—C10—N1	126.67 (11)	C9—C8—H8B	109.00
Se1—C10—N1^i^	126.67 (11)	H8A—C8—H8B	108.00
N1—C10—N1^i^	106.66 (17)	N1—C9—H9A	109.00
N1—C11—C12	132.1 (2)	N1—C9—H9B	109.00
N1—C11—C11^i^	106.54 (18)	C8—C9—H9A	109.00
C11^i^—C11—C12	121.4 (2)	C8—C9—H9B	109.00
C11—C12—C13	117.3 (2)	H9A—C9—H9B	108.00
C12—C13—C13^i^	121.4 (3)	C11—C12—H12	121.00
C2—C1—H1	119.00	C13—C12—H12	121.00
C6—C1—H1	119.00	C12—C13—H13	119.00
C1—C2—H2	120.00	C13^i^—C13—H13	119.00
C3—C2—H2	120.00		
			
C11—N1—C10—Se1	−179.91 (16)	C3—C4—C5—C6	−2.0 (6)
C9—N1—C10—N1^i^	178.5 (2)	C4—C5—C6—C1	1.3 (5)
C11—N1—C10—N1^i^	0.1 (2)	C4—C5—C6—C7	−177.0 (3)
C9—N1—C11—C12	2.2 (4)	C1—C6—C7—C8	130.7 (3)
C10—N1—C11—C12	−179.4 (3)	C5—C6—C7—C8	−51.1 (4)
C9—N1—C11—C11^i^	−178.7 (2)	C6—C7—C8—C9	−178.1 (2)
C10—N1—C11—C11^i^	−0.2 (2)	C7—C8—C9—N1	−177.9 (2)
C9—N1—C10—Se1	−1.5 (3)	N1—C11—C12—C13	179.5 (3)
C10—N1—C9—C8	−88.2 (3)	C11^i^—C11—C12—C13	0.5 (4)
C11—N1—C9—C8	90.0 (3)	N1—C11—C11^i^—N1^i^	0.3 (2)
C6—C1—C2—C3	−1.1 (6)	N1—C11—C11^i^—C12^i^	179.6 (2)
C2—C1—C6—C5	0.2 (5)	C12—C11—C11^i^—N1^i^	179.6 (2)
C2—C1—C6—C7	178.5 (3)	C12—C11—C11^i^—C12^i^	−1.2 (4)
C1—C2—C3—C4	0.5 (6)	C11—C12—C13—C13^i^	0.9 (5)
C2—C3—C4—C5	1.0 (6)	C12—C13—C13^i^—C12^i^	−1.6 (5)

Symmetry codes: (i) *y*, *x*, −*z*+1.

### Hydrogen-bond geometry (Å, °)

**Table:**

*D*—H···*A*	*D*—H	H···*A*	*D*···*A*	*D*—H···*A*
C9—H9B···Se1	0.97	2.92	3.310 (3)	105
